# Bub1 targeting to centromeres is sufficient for Sgo1 recruitment in the absence of kinetochores

**DOI:** 10.1007/s00412-016-0592-7

**Published:** 2016-04-26

**Authors:** Samantha J. Williams, Ariane Abrieu, Ana Losada

**Affiliations:** 1 Chromosome Dynamics Group, Molecular Oncology Programme, Spanish National Cancer Research Centre (CNIO), Melchor Fernández Almagro 3, 28029 Madrid, Spain; 2 Université Montpellier, CRBM, 34293 Montpellier, France

**Keywords:** Kinetochore, Cohesin, Xenopus, Chromosome

## Abstract

Centromeric chromatin containing the histone H3 variant centromere protein A (CENP-A) directs kinetochore assembly through a hierarchical binding of CENPs, starting with CENP-C and CENP-T. Centromeres are also the chromosomal regions where cohesion, mediated by cohesin, is most prominently maintained in mitosis. While most cohesin dissociates from chromosome arms in prophase, Shugoshin 1 (Sgo1) prevents this process at centromeres. Centromeric localization of Sgo1 depends on histone H2A phosphorylation by the kinase Bub1, but whether additional interactions with kinetochore components are required for Sgo1 recruitment is unclear. Using the Xenopus egg cell-free system, we here show that both CENP-C and CENP-T can independently drive centromeric accumulation of Sgo1 through recruitment of Bub1 to the KNL1, MIS12, NDC80 (KMN) network. The spindle assembly checkpoint (SAC) kinase Mps1 is also required for this pathway even in the absence of checkpoint signaling. Sgo1 recruitment is abolished in chromosomes lacking kinetochore components other than CENP-A. However, forced targeting of Bub1 to centromeres is sufficient to restore Sgo1 localization under this condition.

**Electronic supplementary material:**

The online version of this article (doi:10.1007/s00412-016-0592-7) contains supplementary material, which is available to authorized users.

## Introduction

Sister chromatid cohesion mediated by the cohesin complex protects cells against aneuploidy (Losada 2014). Cohesin is loaded on chromatin in G1 and cohesion is established in S phase. At the onset of mitosis, most cohesin dissociates from chromatin in a process that requires phosphorylation of cohesin and some of its associated factors by Plk1, Aurora B, and Cdk1 (Liu et al. 2013; Losada et al. 2002; Nishiyama et al. 2013; Sumara et al. 2002). Importantly, a population of cohesin remains on the condensed chromosomes, mostly at centromeres, to prevent precocious separation of sister chromatids until proper alignment of the chromosome in the metaphase plate is achieved. This population is kept in a hypophosphorylated state by the Shugoshin (Sgo) 1-PP2A complex (Kitajima et al. 2006; McGuinness et al. 2005; Rivera and Losada 2009). Sgo1 also promotes centromeric accumulation of the chromosomal passenger complex (CPC), which is required for Aurora B to regulate biorientation (Kawashima et al. 2007; Yamagishi et al. 2010). In turn, Sgo1 delocalizes to chromosome arms if the CPC is inactive (Boyarchuk et al. 2007; Rivera et al. 2012). How Sgo1 targets the centromere specifically is not completely understood. The kinase Bub1 phosphorylates threonine 120 of histone H2A (phosphoH2A hereafter) in the chromatin underneath to create a signal that Sgo1 likely recognizes (Kawashima et al. 2010; Kitajima et al. 2005; Liu et al. 2015; Tang et al. 2004). Bub1 heterodimerizes with Bub3, which in turn recognizes Met–Glu–Leu–Thr (MELT) motifs in the outer kinetochore component Knl1 once they become phosphorylated by Mps1 (Krenn et al. 2012; Vleugel et al. 2013; Yamagishi et al. 2012). Whether additional components of the kinetochore contribute to Sgo1 recruitment is unclear. In budding yeast, it has been proposed that Chl4/CENP-N could indeed promote Sgo1 recruitment through a direct interaction between the two proteins (Hinshaw and Harrison 2013).

Centromeric chromatin contains nucleosomes carrying the histone H3 variant centromere protein A (CENP-A) in addition to canonical H3 nucleosomes (Fachinetti et al. 2013; Fukagawa and Earnshaw 2014). Kinetochore assembly occurs through a hierarchical process that involves two somewhat parallel pathways directed by CENP-C, which recognizes CENP-A, and CENP-T, which recognizes H3 nucleosomes, within centromeric chromatin (Basilico et al. 2014; Carroll et al. 2010; Gascoigne et al. 2011; Hori et al. 2008; Kim and Yu 2015; Logsdon et al. 2015). In Drosophila, the CENP-C pathway appears to be sufficient for kinetochore assembly (Drinnenberg et al. 2014; Przewloka et al. 2011). We recently found that these two pathways can be reconstituted in Xenopus egg extracts (Krizaic et al. 2015) and decided to employ this cell-free system to dissect the cross talk between centromeric cohesion and kinetochore assembly.

## Results and discussion

### Similar contribution of CENP-C and CENP-T to Sgo1 recruitment

**Fig. 1:**
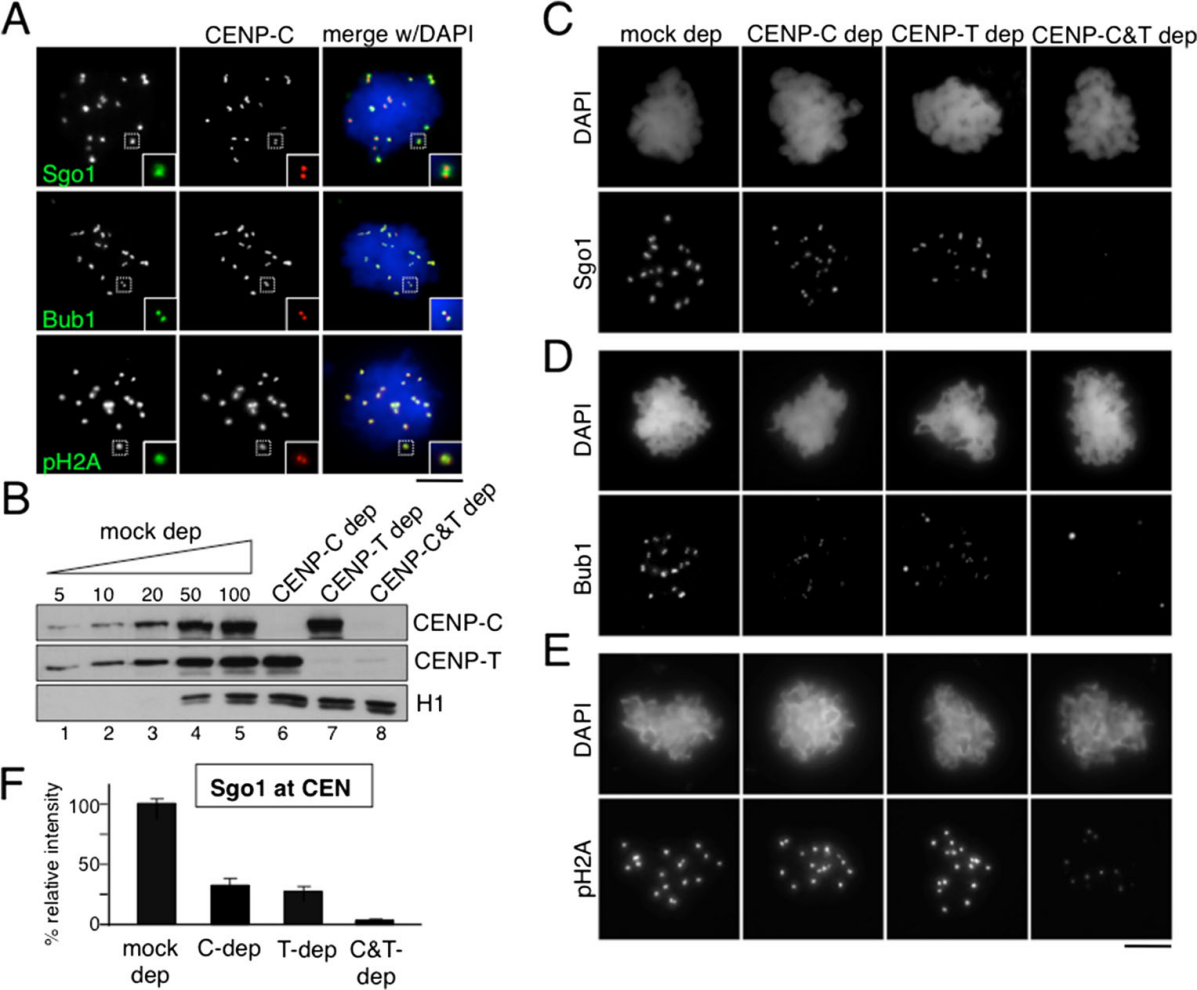
Both CENP-C and CENP-T promote Sgo1 recruitment to centromeres. **a** Representative examples of replicated chromosomes assembled in the egg extracts and stained with the indicated antibodies. *Insets* highlight an individual centromere within each chromosome mass. Scale bar, 10 μm. **b** Immunoblot analysis of the extracts used to assemble the chromosomes shown in (**c**–**e**). Increasing amounts of mock-depleted CSF extract, expressed as percentage, and aliquots of extracts depleted with specific antibodies, as indicated, were analyzed side by side to estimate the extent of each depletion. H1 served as a loading control. **c**–**e** Representative examples of chromosomes assembled in the indicated extracts and stained with antibodies against Sgo1 (**c**), Bub1 (**d**), and phosphoH2A (pH2A) (**e**). For validation of the pH2A antibody see Online Resource 1. Scale bar, 10 μm. **f** Quantification of average fluorescence in centromere pairs per nucleus (chromosome mass), expressed as a percentage of the average obtained in mock depleted extracts. Bars represent mean ± SD. More than ten nuclei were measured per condition in each of the three independent experiments

**Fig. 2:**
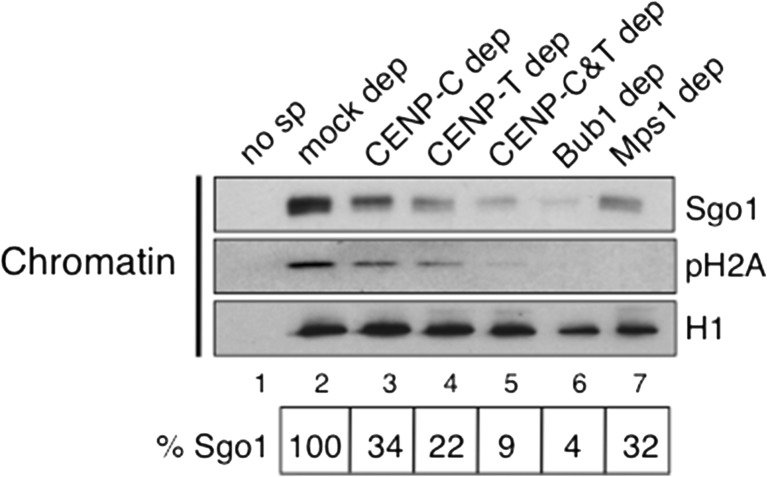
Reduced Sgo1 recruitment to chromatin in the absence of CENP-C, CENP-T, Bub1, or Mps1. Immunoblot analysis of chromatin fractions from replicated chromosomes assembled in the indicated extracts and purified by centrifugation through a sucrose cushion (lanes 2–7). Chromatin purified in the same way from a mock assembly reaction without sperm served as control (no sp, lane 1). Histone H1 was used as loading control. Quantification of the Sgo1 signals, normalized to the H1 signals, and expressed relative to the Sgo1 signal in the chromatin obtained in the mock-depleted extract

Mitotic chromosomes with paired sister chromatids and sister kinetochores can be assembled from sperm chromatin in Xenopus egg extracts (Losada et al. 1998). The sperm chromatin contains CENP-A but all the other kinetochore proteins have to be recruited de novo (Bernad et al. 2011; Krizaic et al. 2015; McCleland et al. 2003; Milks et al. 2009). For chromosome assembly, cytostatic factor (CSF) extracts prepared from eggs arrested in meiosis II are first released to interphase by addition of calcium and then sperm chromatin is added. After incubation for 90 min to allow DNA replication to take place, more CSF extracts or cyclin B are added to drive extracts back to mitosis. The resulting mitotic chromosomes are often not individualized but appear entangled forming a chromosome mass in which accumulation of Sgo1 can be observed on the chromatin surrounding the kinetochores labeled by CENP-C (Fig. 1a, top). Bub1 and phosphoH2A staining can also be detected at kinetochores and the surrounding chromatin, respectively (Fig. 1a, middle and bottom, respectively; see also Online Resource 1 for characterization of phosphoH2A antibody). To test whether Sgo1 recruitment to centromeres in mitosis depends on kinetochore assembly mediated by CENP-C or CENP-T, replicated chromosomes were assembled in extracts from which these factors had been depleted (Fig. 1b). Immunofluorescence analysis showed a partial decrease in Sgo1 staining in the absence of either protein and a complete absence when the two were depleted simultaneously (Fig. 1c, top; quantification in Fig. 1f). A comparable reduction of Bub1 kinetochore signals was observed in chromosomes assembled in the absence of CENP-C or CENP-T, and no signal could be detected in the doubly-depleted chromosomes (Fig. 1d, middle; quantification in Online Resource 2). These reduced amounts of Bub1 could still generate a phosphoH2A signal that, in contrast, was not seen in chromosomes lacking both CENP-T and CENP-C or lacking Bub1 (Fig. 1e, bottom; quantification in Online Resource 2). Similar results were obtained by immunoblot analyses of the corresponding chromatin fractions (Fig. 2, lanes 1–5).

We recently reported that the amount of CENP-T present at centromeres in mitotic chromosomes assembled in extracts lacking CENP-C was reduced to around 20 % of its level in chromosomes from control extracts. Despite this reduction, the KMN network components Ndc80 and Mis12 were targeted to kinetochores with similar efficiency in chromosomes from CENP-C- or CENP-T-depleted extracts (Krizaic et al. 2015). Since Knl1 binds Mis12, we suspect that comparable amounts of Knl1 may be present in chromosomes lacking CENP-C or CENP-T. An antibody against Xenopus Knl1 is not available at the moment to confirm this. In any case, our results suggest that the two pathways of kinetochore assembly driven by CENP-C and CENP-T make similar contributions to the recruitment of Bub1, the generation of the phosphoH2A signal, and the accumulation of Sgo1 at centromeric chromatin in mitosis.

### Mps1 is required for Bub1 and Sgo1 recruitment to kinetochores

**Fig. 3:**
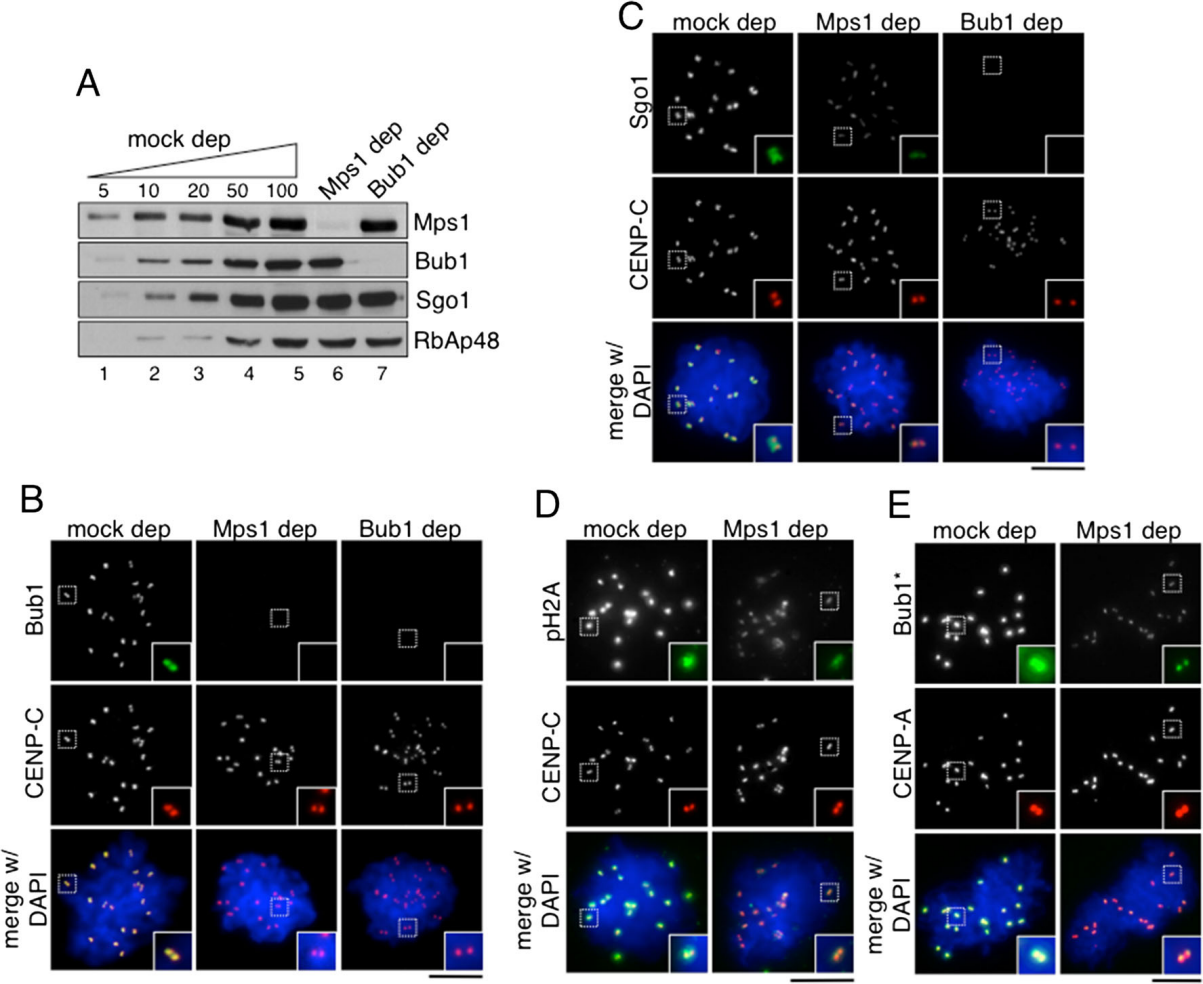
Mps1 is required for Bub1 and Sgo1 targeting to the centromere. **a** Immunoblot analysis of the extracts used in (**b**–**e**) to estimate the efficiency of the depletion and to confirm that these depletions did not alter Sgo1 levels in the soluble extracts. RbAp48 was used as loading control. **b**–**e** Representative examples of chromosomes assembled in extracts lacking Mps1 or Bub1, as well as in mock-depleted extracts, and stained with antibodies against Bub1 (**b**), Sgo1 (**c**), pH2A (**d**), or a Bub1 antibody (Bub1*) different from the one used in (**b**) and Fig. 1. CENP-C or CENP-A was used to label centromeres. *Insets* highlight an individual centromere pair within each chromosome mass. Scale bar, 10 μm

Several studies underscore the importance of the kinase Mps1 for regulation of kinetochore microtubule attachment and the spindle assembly checkpoint (SAC) (Abrieu et al. 2001; Hiruma et al. 2015; Ji et al. 2015). Mps1 has been shown to phosphorylate the MELT motifs in Knl1 and thereby promote Bub3/Bub1 recruitment (Vleugel et al. 2015; Yamagishi et al. 2012). Taking advantage of the fact that the SAC is not at work in the Xenopus egg extract under our experimental conditions, we decided to address the role of Mps1 in Sgo1 targeting independent of checkpoint signaling. Chromosomes assembled in extracts depleted of Mps1 to less than 5 % of its normal levels (Fig. 3a) show undetectable Bub1 at kinetochores (Fig. 3b). Surprisingly, however, reduced amounts of Sgo1 could still be observed at the centromeres of these chromosomes, but not of chromosomes from extracts lacking Bub1 (Fig. 3c). Immunoblot analysis of chromatin fractions obtained from these extracts confirmed these observations (Fig. 2, lanes 6–7). Staining with phoshoH2A antibody showed reduced but clearly detectable signals on the Mps1-depleted chromosomes (Fig. 3d), unlike Bub1-depleted chromosomes (Online Resource 1). This result suggested that the little amount of Mps1 remaining in the extract after depletion was sufficient to allow recruitment of a small fraction of Bub1, undetected with our antibody, in turn capable to generate a phosphoH2A signal required for Sgo1 targeting. In fact, staining with a different antibody against Bub1 that produces stronger signals could detect some Bub1 at kinetochores in Mps1-depleted extracts (Fig. 3e). Alternatively, this small fraction of Bub1 could be recruited to Knl1 independent of MELT motif phosphorylation by Mps1. In any case, when we combined the Mps1 depletion with impaired recruitment of the KMN network by depletion of either CENP-C or CENP-T, Sgo1 signals disappeared (Online Resource 3). All together, these results indicate that even in the absence of SAC signaling, Mps1 is required for efficient Bub1 and Sgo1 targeting to centromeres through the KMN network.

### Forced targeting of Bub1 to centromeres rescues Sgo1 targeting in the absence of kinetochore assembly

**Fig. 4:**
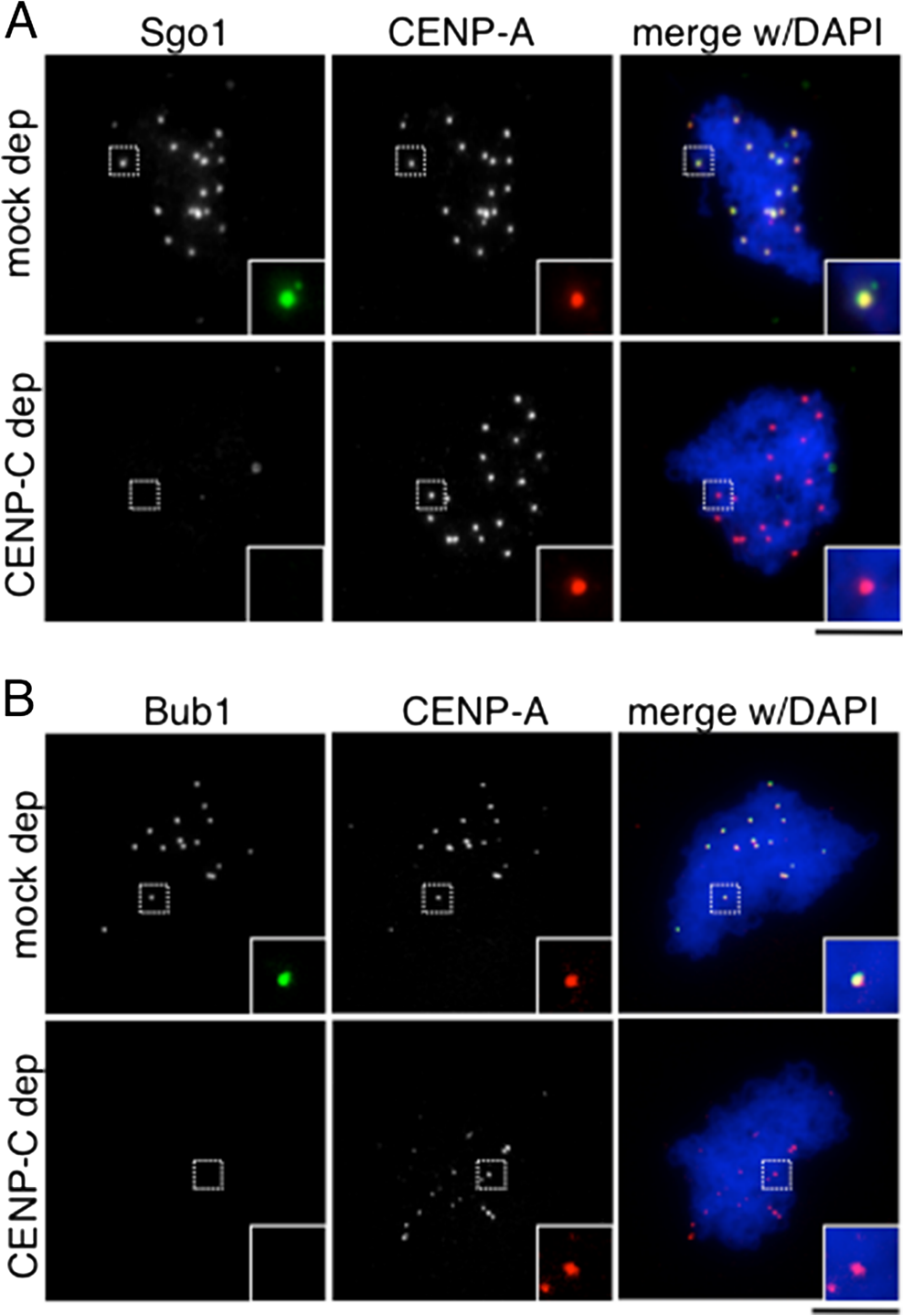
Complete absence of Bub1 and Sgo1 in CSF chromosomes lacking CENP-C. **a**, **b** Depletion of CENP-C is sufficient to fully prevent Sgo1 (**a**) and Bub1 (**b**) recruitment to centromeres of chromosomes assembled in CSF extracts. Note that these chromosomes are not replicated and therefore contain each a single kinetochore labeled by CENP-A. Scale bar, 10 μm

**Fig. 5:**
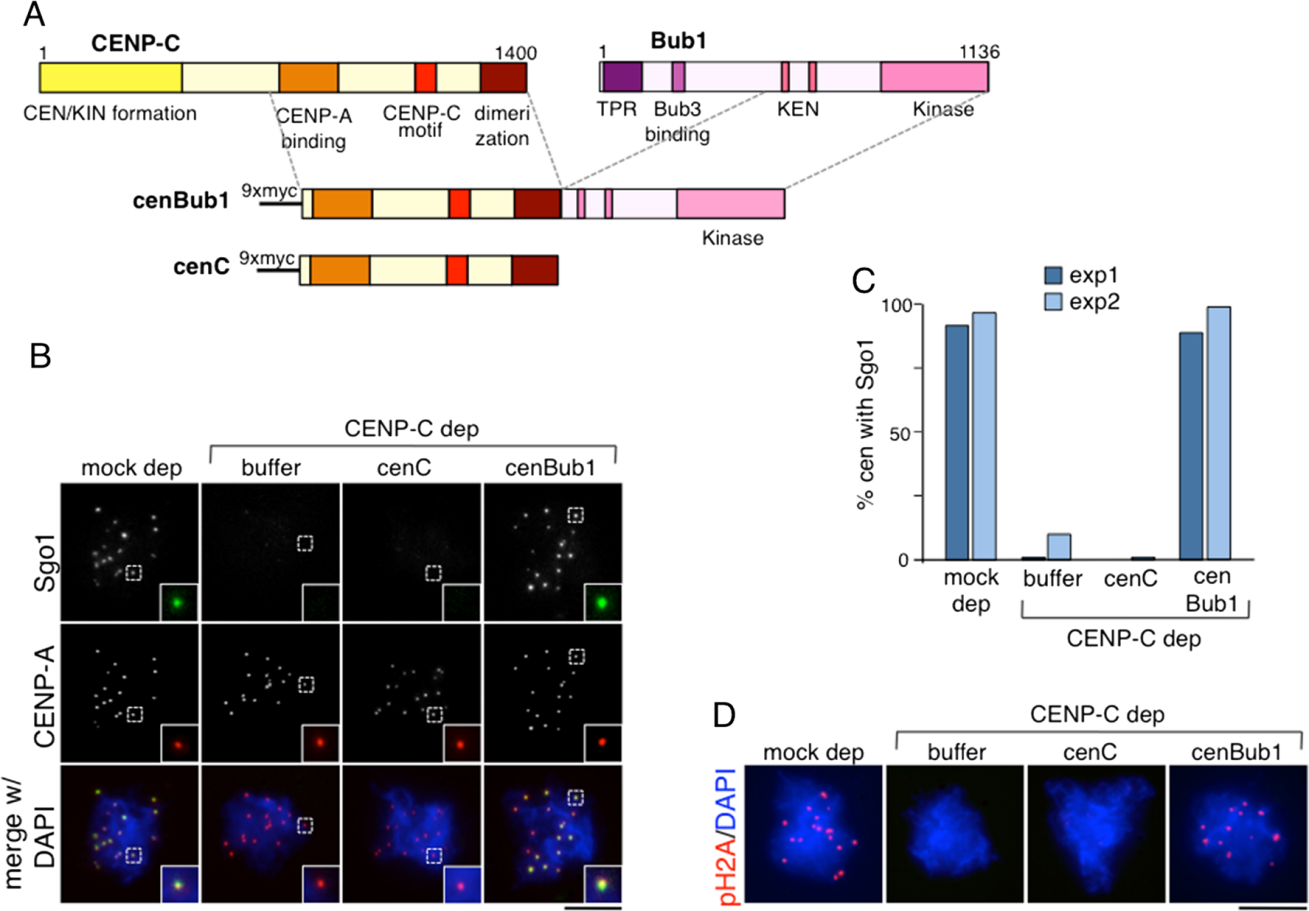
Forced targeting of Bub1 to the centromere rescues Sgo1 targeting in the absence of kinetochores. **a** Schematic representation of the constructs used in (**b**–**d**). **b** Representative examples of chromosomes assembled in mock-depleted extracts and in CENP-C-depleted extracts supplemented with buffer, cenC, or cenBub1 and stained with the indicated antibodies. *Insets* highlight an individual centromere within each chromosome mass. Scale bar, 10 μm. **c** Quantification of the number of chromosome masses showing Sgo1 staining at centromeres in two different experiments (expressed as percentage). More than 120 centromeres were scored per condition in each experiment. **d** Chromosomes assembled as in (**b**) stained with pH2A and DAPI. Scale bar, 10 μm

Previous results in budding yeast have suggested that Chl4/CENP-N interacts with Sgo1 and contributes to its recruitment (Hinshaw and Harrison 2013). We therefore asked whether kinetochore proteins promote Sgo1 accumulation at centromeres exclusively through Bub1 targeting or may contribute more directly to this recruitment. To answer this question, we devised an experiment in which Bub1 could be forced to target the centromeres in the absence of kinetochore assembly. To simplify the experiment, we performed it in CSF extracts in which we previously reported that only the CENP-C pathway of kinetochore assembly exists (Krizaic et al. 2015). Depletion of CENP-C is sufficient to fully prevent Bub1 and Sgo1 recruitment to the centromeres of CSF-assembled chromosomes (Fig. 4). In human cells, forced targeting of factors to centromeres employs CENP-B, a protein that recognizes a sequence on centromeric alphoid satellite DNA (Liu et al. 2009). Unfortunately, a Xenopus CENP-B homolog has not been identified. We therefore fused the kinase domain of Bub1 to the C-terminal half of CENP-C, which is able to target the centromere but unable to recruit outer kinetochore components (Fig. 5a; Milks et al. 2009). We first confirmed that the chimeric protein (“cenBub1” in Fig. 5) and the C-terminal half of CENP-C used as control (“cenC”), both tagged with myc, translated in vitro, could target the centromeres in the absence of endogenous CENP-C but could not recruit Mis12, Ndc80, or Mps1 (Online Resource 4). Next, we checked for Sgo1 recruitment to centromeres. Addition of the chimeric cenBub1 kinase to CENP-C-depleted extracts restored Sgo1 recruitment to centromeres whereas addition of the C-terminal half of CENP-C, cenC, or buffer, did not (Fig. 5b and quantification in Fig. 5c). As expected, phosphoH2A staining was only observed after addition of cenBub1 to the CENP-C depleted extract (Fig. 5d). Thus, the presence of Bub1 at centromeres in the absence of kinetochore proteins generates the phosphoH2A signal and is sufficient for Sgo1 recruitment.

## Conclusion

We have shown that both CENP-C and CENP-T can independently recruit the KMN network and thereby provide a landing pad for Bub1 in mitosis. CENP-C appears to have a predominant role in kinetochore assembly in human somatic cells (Basilico et al. 2014; McKinley et al. 2015). In the Xenopus cell-free system, depletion of CENP-C also reduces the amount of CENP-T at mitotic centromeres, but the little fraction that is left can recruit as many KMN complexes as CENP-C does in the absence of CENP-T (Krizaic et al. 2015). Similar amounts of Bub1 are then recruited to Knl1 in either condition, with the contribution of Mps1. We have also found that targeting of Bub1 to centromeres is sufficient to recruit Sgo1 to this region in the absence of kinetochore proteins other than CENP-A. Additional pathways such as the one proposed in budding yeast for Chl4/CENP-N (Hinshaw and Harrison 2013) or for HP1 in human cells (Kang et al. 2011; Yamagishi et al. 2008) are not strictly required, at least in this embryonic system, although they may strengthen Sgo1 accumulation in centromeric chromatin provided that histone H2A is phosphorylated by Bub1. In budding yeast, a phosphomimetic H2A mutant (H2A-S121D) cannot rescue Sgo1 targeting in the absence of Bub1, a result that suggests that this kinase has another yet unidentified substrate important for Sgo1 recruitment (Nerusheva et al. 2014). According to our results, this substrate is unlikely to be a kinetochore protein.

## Materials and methods

### Antibodies

We raised a rabbit polyclonal antibody against phosphorylated threonine 120 of *Xenopus laevis* histone H2A by injecting rabbits with the phosphopeptide peptide CLLPKK(pT)ESAKS (Innovagen, SE). Other antibodies used in this study have been described before: Xenopus CENP-C and CENP-T (Krizaic et al. 2015); embryonic histone H1, RbAp48 (Bernad et al. 2011); Xenopus Bub1, Sgo1, CENP-A (Rivera and Losada 2009); Mps1 (Morin et al. 2012); Mis12 and Ndc80 (a generous gift of P.T. Stukenberg; Emanuele et al. 2005); anti-myc (clone 9E10).

### Immunodepletion and add-back experiments

For immunodepletion, antibodies were bound to Protein A Dynabeads (Life Technologies) or PureProteome magnetic beads (Millipore). Depletion of 100 μl of extract required one (CENP-C, Mps1) or two rounds (CENP-T, Bub1) of incubation with 50 μl of beads bound to 30 μg (PureProteome) or 18 μg (Dynabeads) of antibody. For the add-back experiments in Fig. 5 and Online Resource 4, the fragment of Xenopus CENP-C coding for amino acids 712–1400 was cloned into pCS2+*myc* vector (cenC), and then the kinase domain of Xenopus Bub1 (amino acids 490–1136) was added (cenBub1). The corresponding *myc*-tagged proteins were produced with TNT Quick Coupled Transcription/Translation system (Promega). The reticulocyte lysate containing the protein was added to the CENP-C-depleted CSF extract (up to 10 % of extract volume) before addition of the sperm. Full-length Xenopus CENP-C and Bub1 cDNAs were kindly provided by A. F. Straight and R.-H. Chen, respectively.

### Chromatin assembly in Xenopus egg extracts

Cytostatic factor (CSF)-arrested low speed supernatants of Xenopus eggs were prepared in XBE2 buffer (10 mM K-Hepes (pH 7.7), 0.1 M KCl, 2 mM MgCl_2_, 0.1 mM CaCl_2_, 5 mM EGTA, and 50 mM sucrose) as described (Losada et al. 1998). To obtain interphase extracts, cycloheximide and CaCl_2_ were added (100 μg/ml and 0.7 mM, respectively) and incubation proceeded for 30 min at 22 °C. To obtain unreplicated chromosomes, sperm (800–1000 nuclei/μl) was incubated in CSF extracts for 90 min at 22 °C. To obtain replicated chromosomes, sperm was first incubated in interphase extracts for 90 min at 22 °C. Then, an equal volume of CSF extract or 100 nM sea urchin cyclin B (purified from a plasmid kindly provided by T. Hirano) was added to the assembly mixtures. These were incubated for additional 90 min at 22 °C before processing them for analysis by immunofluorescence. Chromatin assembly reactions for immunoblot analysis were carried out in the same way but increasing sperm concentration to 2000 nuclei/μl and using cyclin B for driving entry in mitosis. After assembly samples were diluted 10-fold with XBE2 containing 0.25 % Triton X-100 and left on ice for 10 min before centrifugation through a 1-ml cushion of 30 % sucrose in XBE2 at 9000 rpm for 15 min at 4 °C in a swing-out rotor. After removing the assembly mixture and washing the sucrose interface thoroughly with XBE2, the majority of the cushion was removed and a second spin was carried out in a fixed angle rotor at top speed for 2 min. The remaining cushion was discarded and the pellet was resuspended in 10 μl of 1× SDS sample buffer and analyzed by SDS-PAGE and immunoblot.

### Immunofluorescence

Chromosome assembly mixtures were fixed with ten volumes of 2 % paraformaldehyde in XBE2 containing 0.25 % of Triton X-100 for 10 min and spun down on coverslips through a 5-ml cushion of 30 % glycerol in XBE2 at 6500×*g* for 15 min at 4 °C. After washing, coverslips were blocked overnight in 3 % BSA in TBS-0.1 % Triton X-100. For costaining of Bub1 or Sgo1 (mouse monoclonal antibodies) and CENP-A or CENP-C (rabbit polyclonal antibodies), incubation with 2–5 μg/ml primary antibodies for 2 h was followed by 1–2 h incubation in 1:200 donkey anti-mouse FITC and anti-rabbit Cy3. When using two primary antibodies raised in rabbit, one of them labeled, coverslips were incubated for at least 1 h with 0.2 mg/ml non-immune rabbit IgG before applying either Dylight 594-labeled CENP-C or biotin-labeled CENP-A, the latter followed by incubation with Cy3-strepdavidin for 1 h. After washing, coverslips were stained with DAPI and mounted with Mowiol. Samples were analyzed with a Leica DM6000 microscope. Black and white images were taken with a CCD camera and later processed with Photoshop. The same corrections in intensity and contrast were applied for all the images corresponding to the same staining in different conditions for a given experiment. Only in the case of fluorescently labeled CENP-C, used to mark the position of centromeres but never to score differences in intensity, the images may have been processed differently. Quantification of fluorescence intensity was conducted on unprocessed images using Image J (National Institutes of Health).

## Electronic supplementary material

Below is the link to the electronic supplementary material.

ESM 1 (PDF 5462 kb)
