# Supplementary material for: Bub1 targeting to centromeres is sufficient for Sgo1 recruitment in the absence of kinetochores
Source: Chromosoma. 2016 Apr 26;126(2):279–86. doi: 10.1007/s00412-016-0592-7 (PMC5371614; doi:10.1007/s00412-016-0592-7)

Online Resources

Bub1 targeting to centromeres is sufficient for Sgo1 recruitment in the absence of kinetochores

Samantha J. Williams[1], Ariane Abrieu[2] and Ana Losada[1#]

Online Resource 1. Characterization of an antibody against Xenopus H2AphosphoT120.

(A) Comparison of the C-terminal sequence of Xenopus and human H2A. The peptide used as antigen to generate a phosphospecific antibody against Xenopus H2AphosphoT120 is boxed.

(B) Representative examples of chromosomes assembled in mock depleted and Bub1 depleted extracts and stained with antibodies against H2AphosphoT120 [pH2A] and CENP-C. The specificity of the antibody is supported by the fact that it labels centromeres in the control chromosomes but not in those assembled in the absence of the Bub1 kinase. Scale bar, 10 μm.

(C). Immunoblot analysis of extracts and chromatin fractions to show that the signal corresponding to pH2A is present neither in mitotic chromosomes assembled in extracts lacking Bub1 nor in interphase chromatin. In contrast, the mitosis-specific pH3 (Ser10) signal does not depend on Bub1. The left and right parts of the top blot separated by a dotted line correspond to different exposures.

Online Resource 2. Reduced levels of Bub1 and pH2A after depletion of CENP-C and CENP-T. Quantification of average fluorescence in centromere pairs per nucleus (chromosome mass), expressed as a percentage of the average obtained in mock depleted extracts. Bars represent mean± SD. For Bub1 signal quantification, at least 35 nuclei from three independent experiments were measured, with the exception of CENP-C&T depletion condition for which 23 nuclei from two experiments were used. For pH2A signal quantification, more than 20 nuclei from two independent experiments were measured.

Online Resource 3. Codepletion of Mps1 and either CENP-C or CENP-T abolishes Sgo1 targeting to the centromere.

(A) Immunoblot analysis of extracts depleted with the indicated antibodies and used for chromosome assembly in (B). RbAp48, loading control.

(B) Representative examples of chromosomes assembled in extracts lacking Mps1 only, Mps1 and CENP-C or Mps1 and CENP-T, and stained with antibodies against Sgo1 and CENP-A to label centromeres. Scale bar, 10 μm.

Online Resource 4. Forced targeting of Bub1 to the centromere rescues Sgo1 targeting in the absence of kinetochores.

(A) Same as Figure 5A. Schematic representation of the constructs used in (B-D).

(B) Immunoblot analysis of the extracts used to assemble the chromosomes shown in Figure 5. Increasing amounts of mock depleted CSF extract expressed as percentage, and aliquots of the depleted extract, without or with the cenC and cenBub1 proteins depicted in Figure 5A, were analyzed side by side. The CENP-C antibody used for the top blot recognizes the N terminus of CENP-C and could not detect the in vitro translated proteins cenC and cenBub1, which were instead detected with an antibody against the myc tag. H1 served as a loading control.

(C) Both cenC and cenBub1, detected by myc, were recruited to centromeres of chromosomes assembled in the CENP-C depleted extracts. Scale bar, 10 μm.

(D) Mis12, Ndc80 and Mps1 could be detected at centromeres of chromosomes assembled in mock depleted CSF extracts, but not in CENP-C depleted extracts supplemented with buffer, cenC or cenBub1. Scale bar, 10 μm.

Online Resource 1

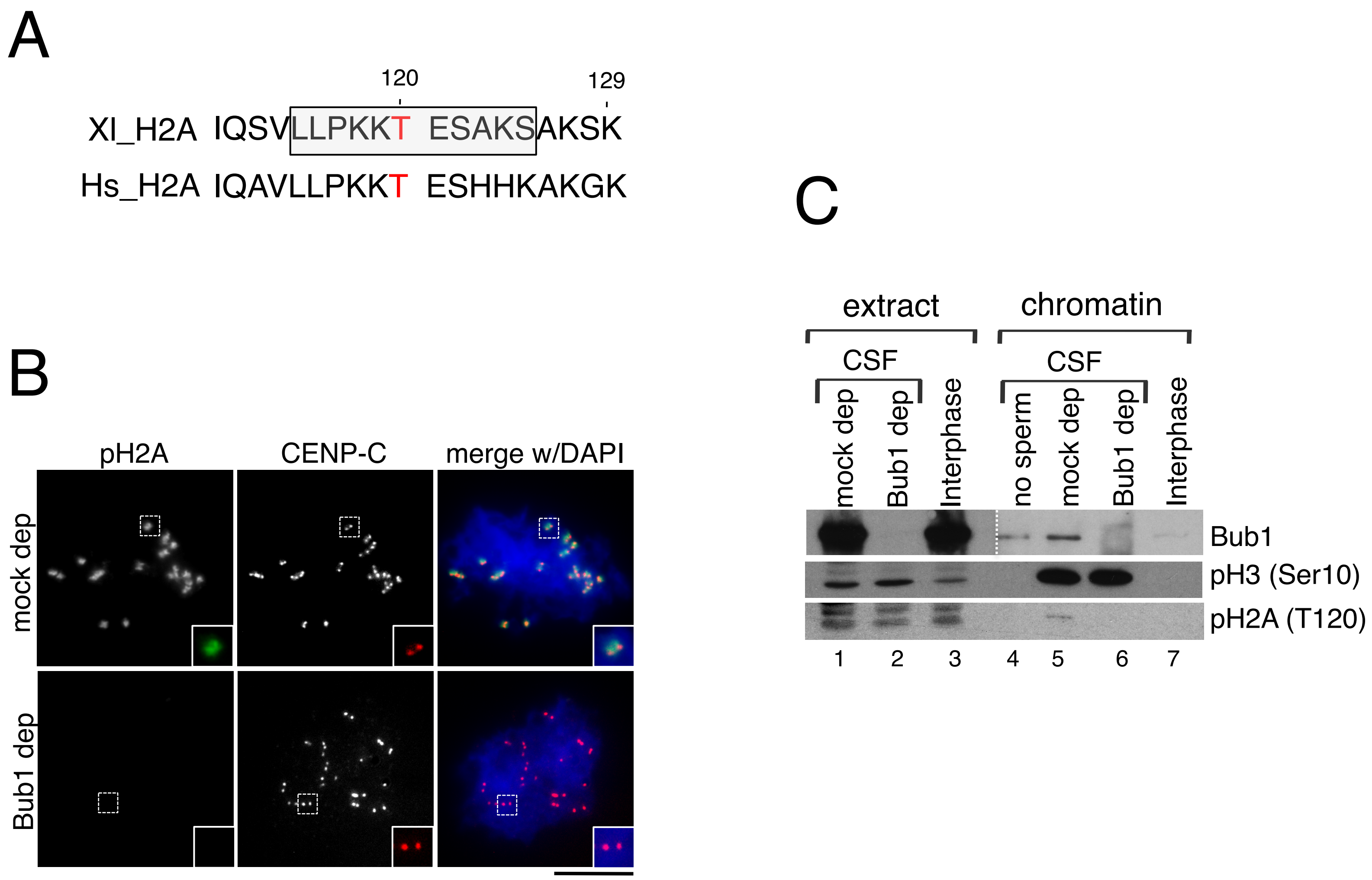

Online Resource 2

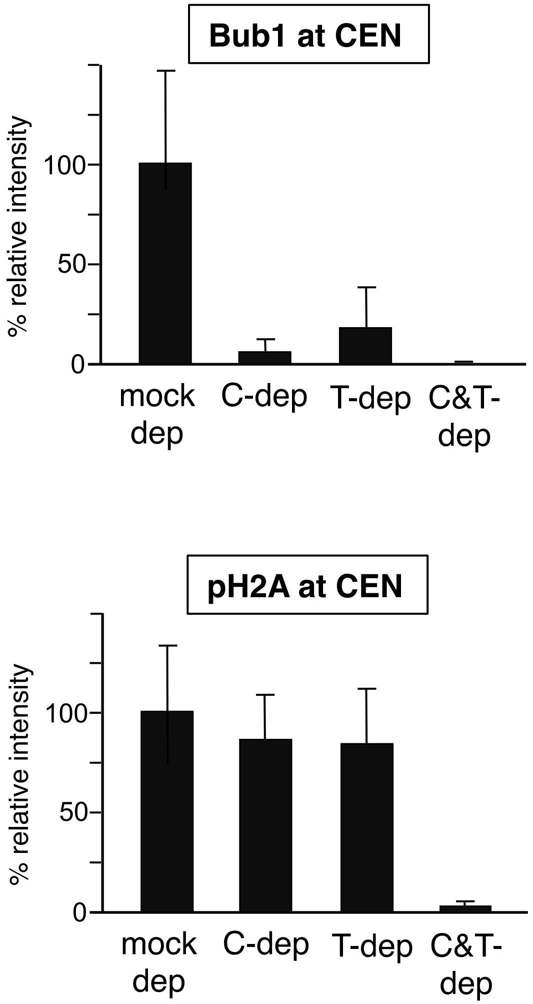

Online Resource 3

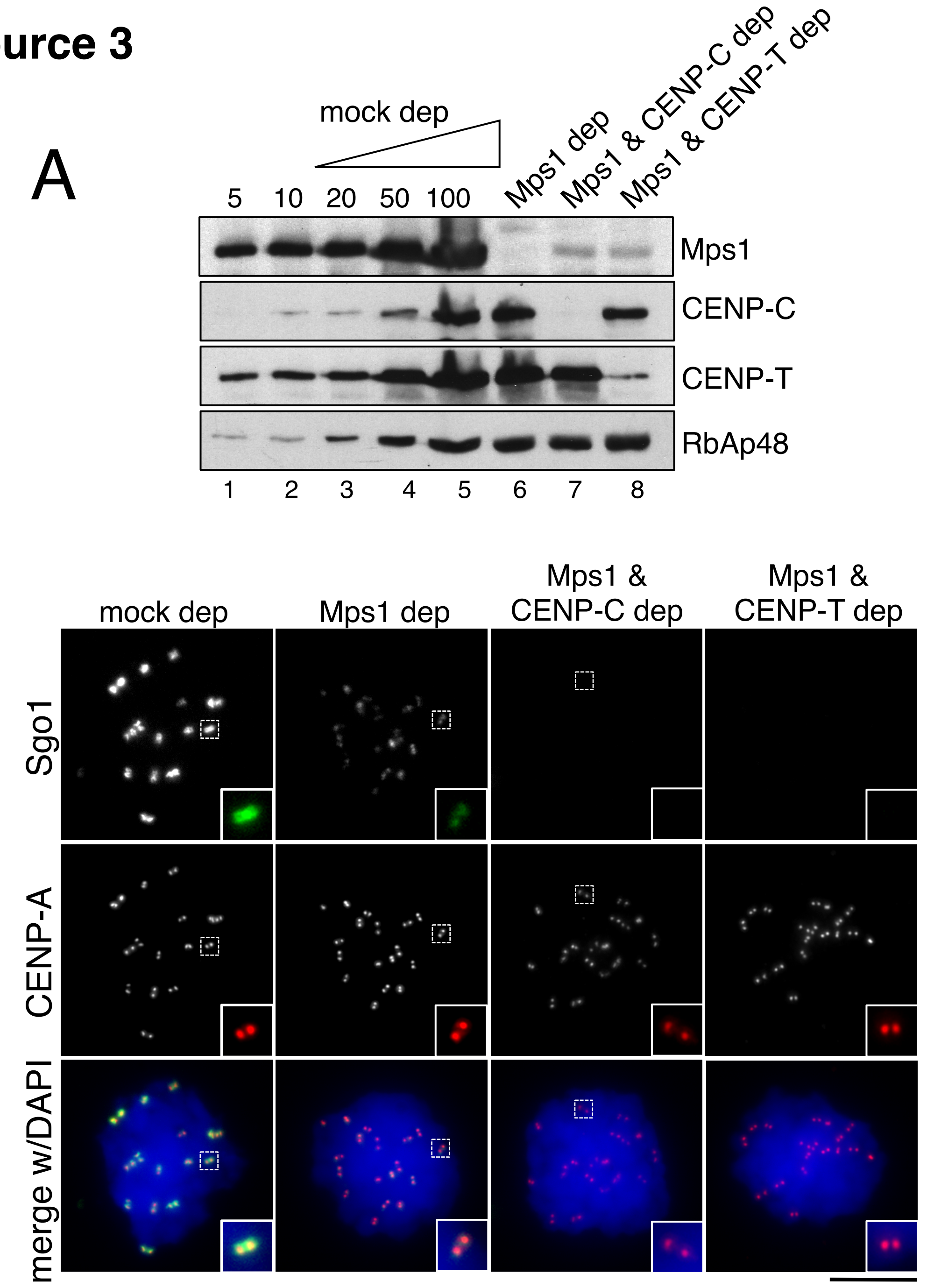

Online Resource 4

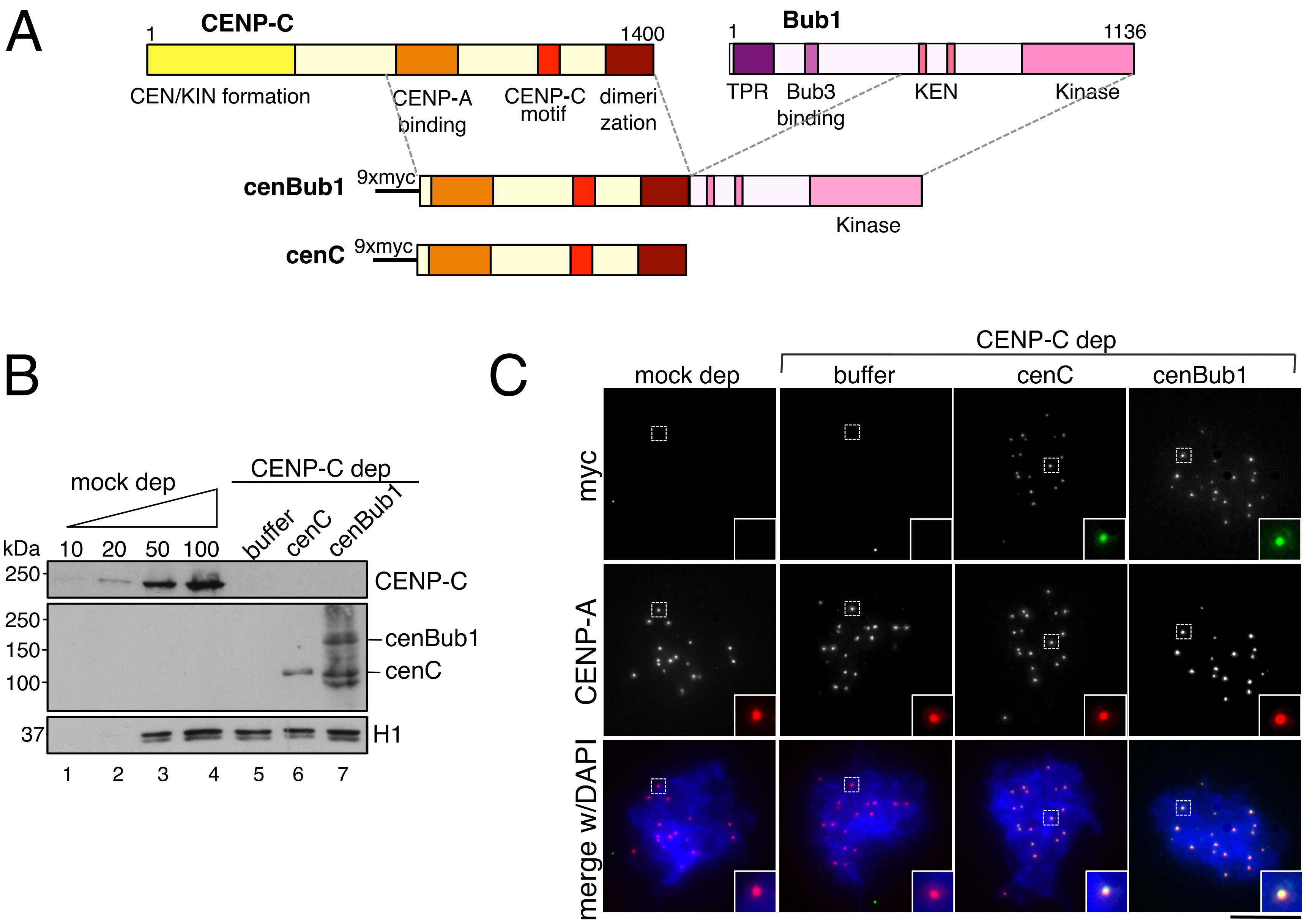

Online Resource 4 (cont)

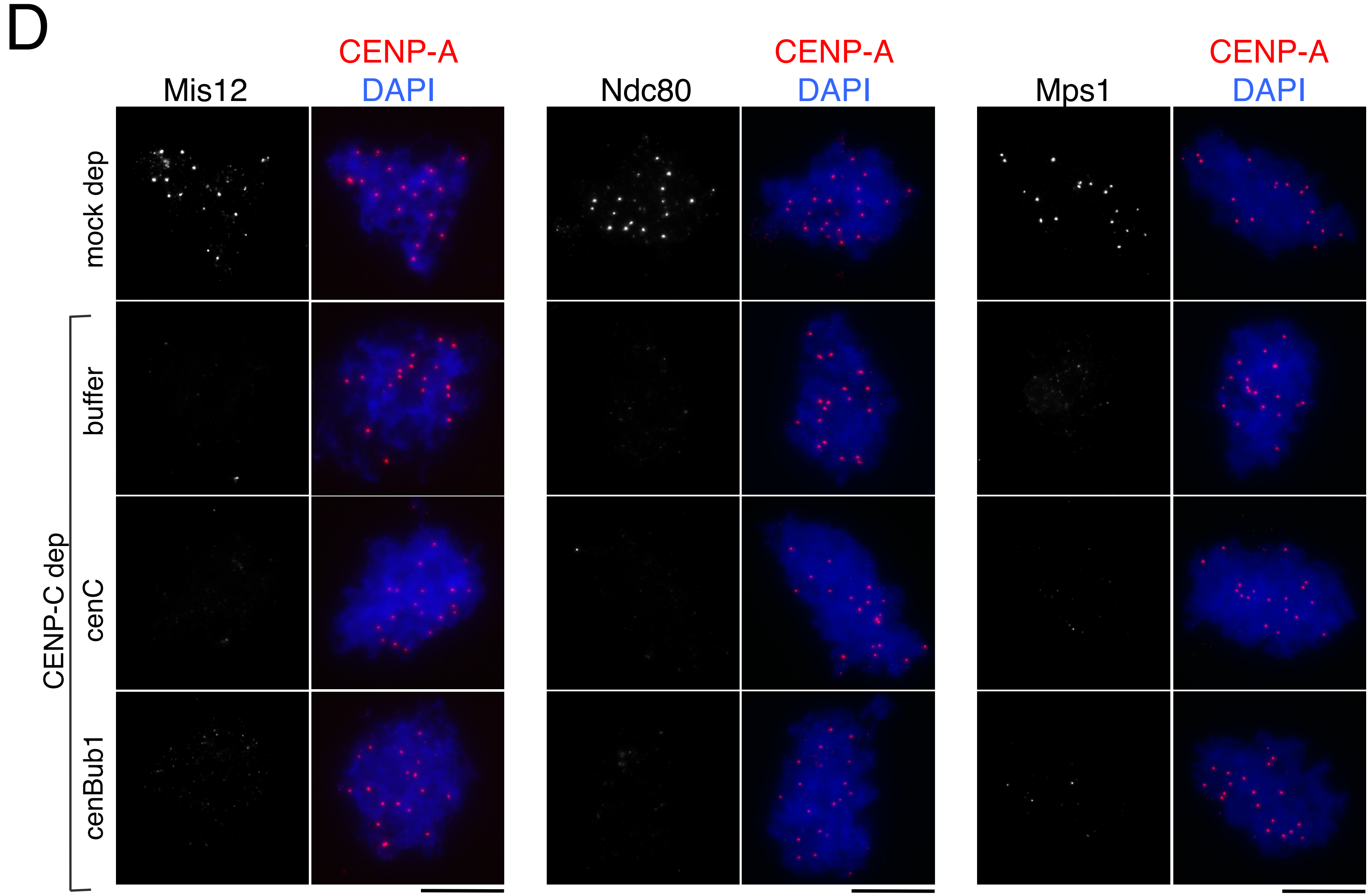

Supplement: Supplementary file 1 — (PDF 5462 kb) [file 412_2016_592_MOESM1_ESM.pdf]
